# Editorial: Well-being of dental professionals and workplace challenges

**DOI:** 10.3389/froh.2025.1699687

**Published:** 2025-10-06

**Authors:** Fawad Javed

**Affiliations:** Department of Orthodontics and Dentofacial Orthopedics, Eastman Institute for Oral Health, University of Rochester, Rochester, NY, United States

**Keywords:** harassment and bullying, workplace, stress, anxiety, dental

The well-being of dental professionals and their teams is not only a matter of personal health but also a cornerstone of the sustainability of the entire profession. Dentistry, like other healthcare disciplines, exposes its workforce to a spectrum of stressors ranging from clinical pressures and job insecurity to systemic inequities and the threat of harassment or violence (1). Such factors, if unaddressed, may lead to burnout, professional disillusionment, and compromised patient care (2, 3). This research topic, focused on “Well-Being of Dental Professionals and Workplace Challenges,” brings together nine studies (Riad et al.; Zhu et al.; Fux-Noy et al.; Queirolo et al.; Queirolo et al.; Javed et al.; Queirolo et al.; Al-Almer et al.; Foláyan et al.) that explore such issues.

A core message of this editorial is the need to transition from merely identifying stressors to offering structured recommendations for action. Dental schools, institutions, and policy makers must implement protective protocols that prioritize occupational safety, including reliable reporting systems for harassment and aggression. Equally critical are structured mental health support schemes, ranging from counseling access to simple stress-management interventions like hypnosis-based techniques that have shown promise for reducing physiological stress responses. Organizational mechanisms must be introduced to combat inequities such as gender discrimination, while academic bodies should create transparent frameworks to prevent editorial bullying and abuse of power. Without such practical measures, editorials risk becoming only descriptive rather than prescriptive, failing to provide the leadership expected of them. Furthermore, it is essential to identify research gaps, for instance, longitudinal studies on the long-term psychological outcomes of workplace aggression, global data on editorial misconduct, and evidence-based models for preventive institutional policies are still scarce and demand urgent attention in the coming years. Equally important is the choice of references that substantiate the argument with depth and relevance. Some cited studies, such as those addressing mask-wearing behaviors among patients, reflect more on societal compliance than on the lived reality of dental professionals (Zhu et al.). For instance, Braquehais et al. (4) provide a nuanced account of acute stress, anxiety, and depression among healthcare workers in Spain during the first COVID-19 wave, emphasizing urgent support needs. The study by Gasparro et al. (5) focused on dentists from Italy, linking fear of infection with job insecurity and depression, underscoring the profession's vulnerability. Likewise, Sarapultseva et al. (6) shed light on psychological distress and post-traumatic stress in Russian dental staff, revealing the enduring mental health risks within the profession.

Another aspect that contributes to the novelty of this editorial lies in its emphasis on overlooked and emerging challenges, particularly those tied to the digital era. The study by Javed et al. highlighted the concept of “editorial bullying” focuses on a previously underrecognized occupational hazard that threatens academic integrity, undermines professional well-being, and exacerbates burnout. By framing scholarly publishing as part of the broader “workplace,” this perspective expands the discourse on professional hazards beyond the operatory or institution to the digital realm. Moreover, by connecting structural inequities, harassment, and digital exploitation, this editorial proposes a comprehensive framework for understanding occupational stress in dentistry. Explicitly stating this contribution, our effort to reconceptualize dental well-being in both physical and digital domains ensure clarity for readers and sets a standard for future discourse.

In conclusion, safeguarding the well-being of dental professionals requires decisive, multi-level interventions. Institutions and policy makers must move beyond rhetoric and implement enforceable protective policies against workplace violence and harassment. Dental schools should embed structured support systems for students and faculty, cultivating resilience early in training. Professional organizations must invest in transparent editorial processes and global monitoring systems to combat bullying and abuse in academic publishing. At the same time, researchers should prioritize filling critical evidence gaps, especially longitudinal and cross-cultural investigations of stress trajectories and recovery patterns. Ultimately, protecting the well-being of dental professionals is both an ethical imperative and a prerequisite for sustaining a resilient and effective oral healthcare workforce. By taking a critical stance, using stronger evidence, and proposing novel frameworks, this editorial aspires not only to summarize existing knowledge but to lead the profession toward solutions.
